# Investigating awareness of the sustainable development and ecological footprint among physiotherapy and rehabilitation students

**DOI:** 10.1038/s41598-025-10399-y

**Published:** 2025-07-09

**Authors:** Duygu Ilgin Gunduz, Goksel Çilga

**Affiliations:** 1 Faculty of Health Sciences, Department of Physiotherapy and Rehabilitation, Manisa Celal Bayar University, Manisa, Turkey; 2 Manisa Celal Bayar Üniversitesi, Sağlık Bilimleri Fakültesi (Uncubozköy Sağlık Yerleşkesi), Uncubozköy M. 5526 Sk. No:8/4, Yunusemre, PK:45030 Manisa, Turkey

**Keywords:** Physiotherapy, Sustainable development, Ecological footprint, Health care, Health occupations

## Abstract

The term environmental physiotherapy is now widely used, and it is emphasised that raising awareness of the environment and health from university education will be important for the transfer of knowledge on this subject into clinical practice in the future. Thus the aim of our study was to determine the awareness of Sustainable Development (SD) and Ecological Footprint (EFP) among students of physiotherapy and rehabilitation department (DPR; 512 − 76.19%). The socio-demographic data and the students’ views on the concepts of SD (8 questions) and EFP (7 questions), and the level of SD and EFP awareness (with SD and EFP Awareness Questionnaires) were recorded. Participants felt that they had insufficient knowledge of these concepts (SD (0–10): 1.65 ± 2.13-EFP (0–10): 2.64 ± 2.34). Although more than 50% of the students had heard of both concepts and had higher awareness scores, they were insufficiently able to define the relationship with their professions (SD: 10.7% and EFP: 12.3%). Physiotherapy students’ awareness of SD and EFP concepts in relation to their professional roles should be improved. It is also important to ensure that they have access to reliable sources of information via social media and the internet, as well as courses to be included in the curriculum.

**Supplementary Information:**

The online version contains supplementary material available at 10.1038/s41598-025-10399-y.

## Introduction

For many years, the causes and solutions to environmental problems have been discussed by professionals working in many areas of our world. However, the consideration of ‘human, environment and health’ issues as parts of a whole has often been overlooked. Healthcare professionals, who have been bystanders to planetary health studies for many years, are now expected to implement sustainable healthcare practices and approaches that reduce the Ecological Footprint (EFP) as quickly as possible^1^. In this transformation process, the importance of health worker education is emphasised^2^, and it is recommended to improve planetary health literacy through field-specific training^3^. The importance of learning about the relationship between sustainability, global environmental change and health, and transferring this knowledge to clinical practice, is particularly emphasised at university level^4^. It also highlights the importance of higher education institutions^5^. Recent scientific literature emphasises the importance of including this subject in the teaching plans of health students from different branches, such as medicine^6–11^, nursing^11–14^, pharmacy^10,15,16^, dentistry^17,18^, psychology^10^, biomedical sciences^10^, occupational therapy^19–22^, audiology^20^, speech and language pathology^20^, physiotherapy and rehabilitation^20,21,23–27^.

Looking at the current situation in physiotherapy and rehabilitation, many clinical practice, research and educational processes include approaches that can positively support “human and environmental health”. However, terms such as green physiotherapy^28^, environmental physiotherapy (EP)^29^, environmental stewardship^30^, planetary health, sustainable healthcare^3,31^, sustainable physiotherapy, sustainability in physiotherapy^32,33^, determining the environmental footprint of physiotherapy practices as part of sustainable healthcare practices and the need to consider this in clinical decision making^32^, the importance of decarbonising healthcare clinics, including physiotherapy clinics in order to achieve sustainability goals^33,34^, active transport, sustainable transport issues and the importance of educating patients and physiotherapists on these issues^35^, the importance of educating physiotherapists in environmental health and sustainable health care^29,36,37^, green gap in EP^25^, environmental scholarship^29^, interconnections between climate change, sustainable development (SD), and global health in physiotherapy practice^24^, The Environmental Physiotherapy Association (EPA) and the EP Agenda concepts have only recently come to the forefront. The EPA is the first international network of physiotherapy clinicians, educators, researchers, and students interested in exploring and advancing the field of EP.The EP Agenda emphasises the inclusion of environmental and sustainability perspectives in physiotherapy curricula as the single most effective action. In this context, the EP Agenda 2023 on SD in physiotherapy education^38^ and the EP Agenda 2027 on SD in physiotherapy education draw attention to the importance of translating the outcomes of these issues into practice^39^. It is stated that the aim is to ensure development by updating the competences and learning outcomes with additions according to the needs of the field^40^. Most importantly, the need for research studies to analyse the current situation for the benefit of physiotherapy in the planning and implementation phases of the professional training to be organised has been emphasised. In addition, it is stated that physiotherapy educational institutions should start thinking and talking about the relationship between human health, the environment and physiotherapy, making the process visible and, more importantly, involving students in this conversation, even suggesting that students’ opinions should be taken into account in the creation of educational curricula^38^. Huss et al. mentioned the importance of the self-awareness and motivation of university educated students in understanding the relationship between sustainability, global environmental change and health, and translating this into clinical practice^4^. In addition, the importance of the issue in terms of global and public health is also emphasised^29^. However, we have not found a study that includes the awareness of physiotherapy students on this issue. For this reason, our research was conducted with the aim of investigating the awareness of SD and EFP among physiotherapy and rehabilitation students.

## Materials and methods

### Study population

This cross-sectional descriptive study was conducted on 512 volunteers (76.19%) from a total of 672 students of Manisa Celal Bayar University (MCBU), Health Sciences Faculty (HSF), Department of Physiotherapy and Rehabilitation (DPR) between November and December 2022 according to the inclusion criteria. The entire population was invited to participate in the study in order to reach the highest possible number of participants. Post-hoc power analysis was performed in G*Power (ver. 3.1.9.7, University of Düsseldorf, Germany) according to the results of the Sustainable Development Awareness Questionnaire (SDAQ) for males and females. The effect size for the group means was found to be Cohen’s d = 0.536. The type 1 error was set at 0.05 and the group size was 360 for females and 152 for males. The final result of the power analysis of our study was found to be 1-β = 0.999. Inclusion criteria were being a student continuing education in formal and secondary education programmes of MCBU, HSF, DPR in the academic year 2022–2023, being 18 years or older and volunteering by signing and approving the informed consent form to participate in the study. Exclusion criteria were not meeting the inclusion criteria and not answering any questions despite signing and agreeing to the informed consent form. However, there were no blank forms in the study.

### Data collection

This study adhered to the principles of the Declaration of Helsinki and was approved by the MCBU, Faculty of Medicine, Health Sciences Ethics Committee (Ethics Board Approval Number 23/11/2022 20.478.486/1587). After the students were informed (purpose of the study, methods, possible risks and benefits, protection of personal data and contact information), written consent was obtained that they volunteered to participate in the study, and then data collection was carried out using the data collection form consisting of four parts and the face-to-face interview technique (10–12 min). The first section consisted of sociodemographic data (8 questions), the second section consisted of students’ views on sustainable development (8 questions) and ecological footprint (7 questions), and the last two sections consisted of two separate awareness surveys that assessed students’ awareness of sustainable development (36 questions) and ecological footprint (40 questions).

The first part of the form included 8 questions about students’ socio-demographic characteristics (age (year), gender (female/male), body weight (kg), body height (m), body mass index (weight/height*height = kg/m^2^), current grade (1–4)/current programme (formal/secondary education), internship status (did (face-to-face/online)/did not do).

In second part, students were questioned about SD and EFP through 8 and 7 questions. These questions explored if students had heard of SD and EFP, learned about them in school, knew their aims, saw any relationship to their professions, rated their knowledge level on a scale (0 = very bad, 5 = average, 10 = very good), felt about their knowledge level, sources of information they used, and felt a need for education on these topics^12,20,41^.

The first and second parts of the data collection form were prepared by the researcher through literature review. The developed first draft for the first and second parts of data collection form was evaluated by the researchers for all questions by the researchers to include “Appropriateness of grammar?, The clarity and unambiguity of items?, The correct spelling of words?, The correct structuring of the sentences?, Appropriateness of font size and space?, Legible printout?, Adequacy of instruction on the instrument?, The structure of the instrument in terms of construction and well- thought out format?, Appropriateness of difficulty level of the instrument for the participants?, Reasonableness of items in relation to the supposed purpose of the instrument?” questions. It was aimed to achieve 100% agreement for each question. Modifications and refinements were made according to the comments received to facilitate better understanding and to organise the sequence of questions (Supplementary Table 1)^42^.

In the last two part, the SD and EFP awareness questionnaires included the SDAQ and The Ecological Footprint Awareness Questionnaire (EFPAQ).

The level of SD awareness was assessed using the SDAQ. It consists of 36 items with three sub-dimensions: economic sustainability (1–13), social sustainability (14–22) and environmental sustainability (23–36). Items 1, 8, 10, 24, 30 and 34 are negative. The highest score is 5 and the lowest score is 1 for each question^43^. SDAQ has been analyzed for validity and reliability in different studies on university students and healthcare professionals. Two studies found Cronbach’s alpha values of 0.91 and 0.93 for the whole questionnaire, while the values for the subscales ranged from 0.74 to 0.94^12,43^.While the health sciences faculty students included physiotherapy and rehabilitation department students in the study conducted with university students, physiotherapists were included in the study conducted with healthcare professionals. Although, the samples in the studies consisted of heterogeneous groups, the scale was found to be valid and reliable in both studies. In these studies, the internal consistency Cronbach’s alpha coefficient of the scale was found to be 0.94 and 0.91^44,45^.

The EFPAQ developed by Coşkun and Sarıkaya, featuring 40 items across five sub-dimensions which are food (1–8), transport and housing (9–15), energy (16–27), waste (28–35) and water consumption (36–40), was used to measure EFP awareness in the last part. There is one control question in the questionnaire and scores are calculated by taking means, ranging from 1 (lowest) to 5 (highest), with higher scores indicating higher awareness. Cronbach’s alpha values for the questionnaire were 0.92 overall, with subscale values ranging from 0.55 to 0.87^46,47^. Günal et al. reported similar Cronbach’s alpha values for the questionnaire and its subscales^48^. The EFPAQ scale has been applied to many different sample groups, including studies consisting of university students, young people and healthcare professionals^49–51^. There is no study investigating the validity and reliability of the scale directly on physiotherapists or physiotherapy students. However, the internal consistency of the scale was found to be 0.979 in a study including physiotherapy and rehabilitation students in a university students sample^51^.

### Data analysis

All data collected were entered into Microsoft Excel and checked for errors to ensure accuracy, and then imported into IBM SPSS Statistics 26.0 for statistical analysis of the data. The Kolmogorov-Smirnov test was used to assess the conformity of the quantitative data distribution to a normal distribution. Primarily, descriptive statistical analysis was used to summarise the obtained sample characteristics and results, using frequencies (n) and percentages (%) to represent categorical variables, and minimum and maximum values (min.-max.), means and standard deviations for numerical variables. *p* < 0.05 was accepted as a significant difference in statistical analysis. The difference between SD awareness level and number of resources, EFP awareness level and number of resources between males and females was analysed using the Mann-Whitney U test. SD awareness and number of resources and EFP awareness and number of resources between grades were analysed using the Kruskal-Wallis analysis of variance test. Once a statistically significant result was found, pairwise comparisons were made using the Mann-Whitney U test with Bonferroni correction. While the Mann-Whitney U test was used to find differences between genders on the SDAQ and the EFPAQ and on the sub-dimension scores of these questionnaires, the analysis of variance test was used to find differences between grades. One-way analysis of variance was used for normally distributed variables, and homogeneity of variances was assessed using the Levene test in the analysis of differences between grades. The Kruskal-Wallis test was used for other variables. The Kruskal-Wallis test was used to compare sources of information. According to the results of the Kruskal-Wallis test, the sources of information were compared in pairs using the Mann-Whitney U test. Correlation analysis was used to determine the relationships between the total survey scores and the subscale scores of each grade and gender. The Pearson correlation coefficient was used for normally distributed variables and the Spearman correlation coefficient for non-normally distributed data.

## Results

The study included 512 volunteers from a total of 672 students (20.72 ± 1.82 years). When comparing by gender, the number of sources used by females to obtain information about EFP was higher than that of males (*p* = 0.043). There was a statistically significant difference between the groups in terms of SD (*p* = 0.000) and EFP knowledge level (*p* = 0.000) and number of sources (*p* = 0.000; *p* = 0.002) (Table 1).

**Table 1:** Level of knowledge and number of information sources used according to gender and grade level.

	Gender	Mean	Standard deviation	Median	Minimum	Maximum	Mean rank	Z	U	*p*
Age (years)	F + M	20.7188	1.81831	21	18	31				
	M	20.7566	1.88053	21	18	30				
	F	20.7028	1.79383	21	18	31				
SD Knowledge level (0–10)	F + M	1.6504	2.12727	0	0	10				
	M	1.7566	2.40583	0.50	0	10	256.61	0.011	27344	0.991
	F	1.6056	1.99999	0	0	9	256.46			
SD-number of information source (number)	F + M	0.8047	1.03412	1	0	7				
	M	0.8355	1.17052	1	0	7	254.80	-0.184	27101.5	0.854
	F	0.7917	0.97228	1	0	5	257.22			
EFP knowledge level (0–10)	F + M	2.6348	2.33526	2	0	10				
	M	2.4803	2.50819	2	0	10	242.30	-1.434	25202	0.152
	F	2.7000	2.25875	3	0	10	262.49			
EFP-number of information source (number)	F + M	1.2422	1.13473	1	0	5				
	M	1.1184	1.13309	1	0	5	237.58	-2.028	24483.5	0.043*
	F	1.2944	1.13293	1	0	5	264.49			

	Grade	Mean	Standard deviation	Median	Minimum	Maximum	p	Grade		p
SD knowledge level (0–10)	1	2.3421	2.31894	2	0	10	0.000*	1–3		0.000*
	2	1.6612	2.08388	0	0	9		1–4		0.005*
	3	1.2426	1.85989	0	0	8				
	4	1.4752	2.13000	0	0	8				
SD-number of information source (number)	1	1.1491	1.22100	1	0	7	0.000*	1–2		0.038*
	2	0.7438	0. 0.9177	1	0	5		1–3		0.000*
	3	0.5809	0.83049	0	0	4		1–4		0.015*
	4	0.7943	1.07914	0	0	5				
EFP knowledge level (0–10)	1	3.6140	2.32154	4	0	8	0.000*	1–2		0.007*
	2	2.6446	2.29441	2	0	10		1–3		0.000*
	3	2.0662	2.16094	2	0	10		1–4		0.000*
	4	2.3830	2.31967	2	0	8				
EFP-number of information source (number)	1	1.5263	1.23525	1	0	5	0.002*	1–3		0.001*
	2	1.3140	1.16928	1	0	5				
	3	0.9926	0.93884	1	0	5				
	4	1.1915	1.14589	1	0	5				

*SD* Sustainable development, *EFP* Ecological footprint, *F* Female, *M* Male, *U* Mann Whitneyy U Analysis. *: Statistical significance.

SD and EFP Awareness Questionnaires’ scores were higher in females than males (Table 2) but there was no difference between grades (Supplementary Table 2).

**Table 2:** Sustainable development and ecological footprint awareness level according to gender.

Variable	Group	Mean	Standard deviation	Median	Minimum	Maximum	Mean rank	Z	U	*p*
EFP-food	F + M	3.0547	0.55346	3	1.13	5				
	M	2.9605	0.70629	3	1.13	5	225.22	-3.119	22605.5	0.002*
	F	3.0944	0.46996	3.1250	1.50	4.38	269.71			
EFP-transportation/housing	F + M	3.2508	0.67831	3.2857	1.29	5				
	M	3.0705	0.75789	3	1.29	5	216.02	-4.032	21207	0.000*
	F	3.3270	0.62747	3.2857	1.57	5	273.59			
EFP-energy	F + M	4.1149	0.62995	4.1667	1.33	5				
	M	3.9572	0.68005	4	2	5	222.58	-3.375	22203.5	0.001*
	F	4.1815	0.59612	4.2500	1.33	5	270.82			
EFP-waste	F + M	3.8713	0.70012	3.8750	1.5	5				
	M	3.6628	0.77123	3.7500	1.50	5	216.17	-4.015	21230	0.000*
	F	3.9594	0.64912	4	1.63	5	273.53			
EFP-water consumption	F + M	3.9152	0.75047	4.0000	1	5				
	M	3.8000	0.76790	4	1	5	235.23	-2.123	24127	0.034*
	F	3.9639	0.73869	4	1	5	265.48			
EFP-total	F + M	3.6414	0.51648	3.6423	1.74	5				
	M	3.4902	0.59351	3.5470	1.74	5	217.56	-3.870	21441	0.000*
	F	3.7052	0.46665	3.7025	1.75	4.75	272.94			
SD-economic sustainability	F + M	54.8906	6.75542	56.0	17	65				
	M	53.7105	7.75590	54.5	17	65	232.13	-2.426	23655.5	0.015*
	F	55.3889	6.23039	57.0	25	65	266.79			
SD-social sustainability	F + M	40.7813	5.56785	42.5	9	45				
	M	38.8947	6.78248	40.0	9	45	202.40	-5.475	19136.5	0.000*
	F	41.5778	4.75758	43.0	11	45	279.34			
SD-environmental sustainability	F + M	60.2539	8.12127	62.0	17	70				
	M	56.1316	9.16492	58.0	17	70	176.23	-7.990	15158.5	0.000*
	F	61.9944	6.95108	63.0	20	70	290.39			
SD-total	F + M	155.9258	18.53474	160.0	48	180		-6.405	17566.5	0.000*
	M	148.7368	21.55994	153.0	48	178	192.07			
	F	158.9611	16.19738	163.0	56	180	283.70			

*SD* Sustainable development, *EFP* Ecological footprint, *F* Female, *M* Male, *U* Mann Whitneyy U Analysis. *: Statistical significance.

Students stated that they knew the relationship between the concepts of SD and EFP and their professions by 10.7% and 12.3% respectively. It was found that 2.3% and 5.7% of them considered their level of knowledge about the concept of SD and EFP as adequate (Table 3).

**Table 3:** Encountering the concepts of sustainable development and ecological footprint.

Questions	Group	Yes	No	Undecided
		*n* (%)	*n* (%)	*n* (%)
Have you heard the concept of SD before?	F + M	268 (52.30)	244 (47.70)	
	M	78 (51.30)	74 (48.70)	
	F	190 (52.80)	170 (47.20)	
Do you know what the goals of SD are?	F + M	72 (14.10)	440 (85.90)	
	M	22 (14.50)	130 (85.50)	
	F	50 (13.90)	310 (86.10)	
Has the concept of SD ever been mentioned in any of your physiotherapy and rehabilitation department courses?	F + M	281 (54.90)	231 (45.10)	
	M	83 (54.60)	69 (45.40)	
	F	98 (55.00)	162 (45.00)	
Do you have any idea about the relationship between SD and physiotherapy profession?	F + M	55 (10.70)	457 (89.30)	
	M	21 (13.80)	131 (86.20)	
	F	34 (9.40)	326 (90.60)	
Do you find your level of knowledge about SD sufficient?	F + M	12 (2.30)	427 (83.40)	73 (14.30)
	M	6 (3.90)	122 (80.30)	24 (15.80)
	F	6 (1.70)	305 (84.70)	49 (13.60)
Do you feel the need to receive education about SD?	F + M	265 (51.80)	55 (10.70)	192 (37.50)
	M	65 (42.80)	28 (18.40)	59 (38.80)
	F	200 (55.60)	27 (7.50)	133 (36.90)
Have you heard the concept of EFP before?	F + M	392 (76.60)	120 (23.40)	
	M	108 (71.10)	44 (28.90)	
	F	284 (78.90)	76 (21.10)	
Has the concept of EFP been mentioned in any of your physiotherapy and rehabilitation department courses?	F + M	299 (58.40)	213 (41.60)	
	M	77 (50.70)	75 (49.30)	
	F	222 (61.70)	138 (38.30)	
Do you have any idea about the relationship between EFP and physiotherapy profession?	F + M	63 (12.30)	449 (87.70)	
	M	22 (14.50)	130 (85.50)	
	F	41 (11.40)	319 (88.60)	
Do you find your level of knowledge about EFP sufficient?	F + M	29 (5.70)	365 (71.30)	118 (23.00)
	M	13 (8.60)	102 (67.10)	37 (24.30)
	F	16 (4.40)	263 (73.10)	81 (22.5)
Do you feel the need to receive training on EFP?	F + M	263 (51.4)	68 (13.30)	181 (35.4)
	M	63 (41.4)	33 (21.70)	56 (36.8)
	F	200 (55.6)	35 (9.70)	125 (34.7)

*SD* Sustainable development, *EFP* Ecological footprint, *F* Female, *M* Male.

When looking at the percentages of the distribution of sources that students preferred to learn about the concepts of SD and EFP, the internet, social media and course resources were in the top 3. This distribution varied according to gender, year level and internship status (Table 4).

**Table 4:** Information source preferences about sustainable development and ecological footprint.

	Information Source											
Group	I don’t know.		Radio + Television		Internet		Social Media		Lesson		Other	
Sustainable development	*n*	%	*n*	%	*n*	%	*n*	%	*n*	%	*n*	%
Total	244	47.70	30	5.90	95	18.60	86	16.80	42	8.20	15	2.90
Male	74	48.70	9	5.90	23	15.10	28	18.40	13	8.60	5	3.30
Female	170	47.20	21	5.80	72	20.00	58	16.10	29	8.10	10	2.80
1st Grade	38	33.30	12	10.50	26	22.80	27	23.70	10	8.80	1	0.90
2nd Grade	56	46.30	8	6.60	22	18.20	22	18.20	9	7.40	4	3.30
3rd Grade	79	58.10	7	5.10	22	16.20	19	14.00	4	2.90	5	3.70
4th Grade	71	50.40	3	2.10	25	17.70	18	12.80	19	13.50	5	3.50
Not doing internship	126	42.60	22	7.4	55	18.60	60	20.30	23	7.80	10	3.40
Had an internship	118	54.60	8	3.70	40	18.50	26	12.00	19	8.80	5	2.30
Ecological footprint												
Total	120	23.40	53	10.40	132	25.80	106	20.70	73	14.30	28	5.50
Male	44	28.90	13	8.60	32	21.10	33	21.70	23	15.10	7	4.60
Female	76	21.10	40	11.10	100	27.80	73	20.30	50	13.90	21	5.80
1st Grade	13	11.40	14	12.30	32	28.10	23	20.20	27	23.70	5	4.40
2nd Grade	25	20.70	14	11.60	29	24.00	29	24.00	17	14.00	7	5.80
3rd Grade	40	29.40	10	7.40	39	28.70	30	22.10	9	6.60	8	5.90
4th Grade	42	29.80	15	10.60	32	22.70	24	17.00	20	14.20	8	5.70
Not doing internship	59	19.90	32	10.80	73	24.70	65	22.00	48	16.20	19	6.40
Had an internship	61	28.20	21	9.70	59	27.30	41	19.00	25	11.60	9	4.20

## Discussion

In physiotherapy and rehabilitation field, it has been highlighted in the literature in recent years that there is a strong need for data and research on the concepts of sustainability and EFP. With this research we investigated the level of awareness of today’s students, who will be the physiotherapists of the future. Our results showed that DPR students are aware of the concepts of sustainability and EFP, but their awareness and the level of competence they feel in defining the relationship between these concepts and the field of physiotherapy and rehabilitation is low and their need for education about this relationship is intense.

Previous studies have frequently examined the awareness of medical and nursing students regarding SD and EFP. Şimşek and Erkin found 36.2% of nursing students had heard of the SD, 22% knew the goals of sustainable development (SDGs), 2.5% remembered that this concept was mentioned in a course during their nursing education, but 59.8% could not remember. In addition, 45.7% of the nursing students in this study reported that they were aware of the relationship between the concept of SD and the nursing profession^12^. Dönmez and Yardımcı found that nursing students had high environmental awareness and medium-level sustainable consumption habits, which were moderately related to each other^13^. Kaşıkçı et al. stated that nursing students were aware of the carbon footprint and its effect on human and environmental health at the fundamental level. Additionally, they emphasize the importance of incorporating sustainable practices into the educational programs of future health professionals to promote sustainable practices in personal and professional settings^14^. Niemi et al. found that 63% of nursing and medical faculty students felt that they did not learn enough about the SDGs and Agenda 2030. Additionally, both groups of students thought that they did not have enough information for their future careers^11^. Another study found that medical students had a very low level of knowledge about the SDGs (15.2%)^6^. Borlu et al. reported that 63.6% of medical students had not heard of SD before. However, 47.8% believed that physicians play an active role in achieving the SDGs^8^. Yavaş et al. stated that there is a weak correlation between the ecological footprint awareness score and the ecological footprint among medical students^9^.

The other two student groups that were frequently studied outside of medicine and nursing were dentistry and pharmacy students.Thakar et al. found that 48.2% of the group, which included dentists and students, were aware of “green dentistry,” and 66.8% were willing to consider their carbon footprint in their professional practices. However, 56.9% of the participants stated that they lacked knowledge^18^. On the other hand, Gershberg et al. reported that 83% of dental students were “quite” or “extremely” concerned about environmental sustainability in dentistry. However, 75% of the students reported being “not at all” or “somewhat” knowledgeable^17^. Chen et al. reported that 70% of pharmacy students said environmentally sustainable pharmacy practice (ESPP) was important for their future careers. Meanwhile, 93.9% and 84.1% agreed the pharmacy profession had responsibilities regarding sustainability and climate change, respectively. However, only 28% of students felt that they understood environmental sustainability concepts, including climate change, quite well or extremely well. Only 10% (*n* = 17) of students rated themselves as quite or extremely knowledgeable about ESPP^15^. Ladhar et al. stated that most pharmacy students reported minimal to moderate knowledge of the healthcare system’s and pharmacy’s contribution to climate change and the environment and strategies to reduce this impact. 91% of the students reported that they thought this topic was important for their future pharmacy careers^16^.

As far as physiotherapy is concerned, research often focuses on physiotherapists, and to our knowledge, we have not come across any research that only examines students. According to Chi et al., only 19.4% of physiotherapists considered their knowledge of climate change to be sufficient 83.9% of physiotherapists stated that there is a relationship between climate change and physiotherapy practices, and 79.6% believed that climate change affects patients’ health. 53.1% of physiotherapists believed that they have a role to play in climate change^24^. In a study conducted by Sánchez Ibáñez et al. at 52 physiotherapy clinics in Spain, 33 clinics (63.5%) reported that they were unfamiliar with the concept of SD in physiotherapy. Only six (11.5%) stated that they had heard of the EP Agenda on SD. The study also found that, although most clinics were aware of SD, most did not implement SD protocols sufficiently^52^. Irlam et al. stated that 64.4% of medical and health and rehabilitation sciences students were aware of climate change and 72% were aware of environmental sustainability, but understood very little about these concepts^20^. Olafsdottir and Petursdottir, on the other hand, reported that 56.6% of physiotherapists considered their knowledge level to be intermediate, yet only 7.9% could define the concept. Additionally, the therapists believed that the negative environmental effects of physiotherapy practices should be reduced (42.6%), that the issue of reducing the environmental impact of physiotherapy was relevant (49.1%), that they had a responsibility to raise awareness of this issue among people (19.4%) and their patients (25%), and that their practices were eco-friendly (27.1%). Fifty-point-9% stated that their workplace has an environmental policy, 74.8% stated that they are committed to reducing the environmental impact of their work, and 95.4% stated that they are personally committed to this issue^27^.

In line with these findings, students in our study felt that they were more aware but had inadequate knowledge of the concepts of SD and EFP. They gave themselves very low scores out of 10 points. While over 50% of students had heard of both concepts and remembered being taught about them in at least one vocational course, they could not define their relationship to their occupations. Furthermore, awareness of the relationship between these concepts and professional practice was significantly lower in our study (SD: 10.7%; EFP: 12.3%).

The aforementioned studies reveal that nursing, medicine, dentistry, pharmacy, and physiotherapy students do not feel sufficiently knowledgeable about the basic concepts of “environment, sustainability, climate change, and ecological footprint” related to “environment and health” and the relationship between these concepts and their professional roles, and that they express a need for education even if they have already covered these topics in class. Research examining the situation from the perspective of physiotherapy by evaluating students and physiotherapists also revealed that, although there is awareness of these concepts, there are shortcomings in understanding and defining them and integrating them into clinical practice, and the importance of integrating these topics into education was emphasized. As a result, when compared to previous study results, physiotherapy students were found to perceive themselves as less competent than other professionals in determining the relationship between roles and concepts. This situation suggested that the issue should not remain a theoretical concept, but should be urgently converted into a clinical application. Recently, the concept of “Education for Sustainable Healthcare (ESH)” has emerged in the context of sustainable healthcare practices. This concept involves defining the relationship between the environment and human health, ensuring the environmental sustainability of health systems, and revealing how healthcare professionals’ responsibilities are affected by this issue^53^. It is emphasized at this point that the development of “knowledge, skills, and attitudes” must be achieved during the education of healthcare professionals^1,53,54^. Updates to physiotherapy and rehabilitation education will allow us to reevaluate professional roles in line with current needs. This process requires integrating environmentally conscious physiotherapy practices into a concrete concept that informs environmentally conscious and adequate clinical decision-making, rather than a theoretical concept accompanied by ethical values^54–56^.

On the other hand, it is also important to understand the factors that motivate individuals during the education and transformation process and those that create barriers. In this regard, Murray et al. reported that only 21% of occupational therapy students felt prepared to overcome this challenge thanks to their educational experiences. In contrast, the most common barriers were feeling that no one was listening (38.7%), lack of information (33.9%), lack of information on how to get involved (37.1%), and feeling inadequately qualified (37.1%). The most commonly identified supports were more local opportunities for participation (51.6%), more information about climate change and climate action (50%), and education on how to get involved in climate issues (38.71%)^22^. Olafsdottir and Petursdottir state that among the most important factors motivating physiotherapists to adopt eco-friendly practices, 60.5% cited guidance and 46.5% cited education on the relationship between climate change, physiotherapy, and health. On the other hand, the most important barriers were identified as a lack of information (50%) and a lack of eco-friendly materials/equipment (30.7%)^27^. Chi et al. identified lack of information (51.2%) and lack of time (47.6%) as the most significant barriers. 77.2% of respondents indicated that continuing education in the field of climate change and health was the most important solution^24^.

As a result, while education is among the most important motivating factors in this process, feeling inadequate about the subject is seen as the most significant obstacle. The data from our research is consistent with these factors, suggesting that students are hindered by the obstacle created by their feelings of inadequacy. Unlike our study, these studies address the content of education, the level at which it should be incorporated, the topics to be included, and the preferred methods for lessons and assessment. However, the results of these studies and our study highlight the importance of revealing the relationship between these concepts and professional roles and responsibilities in education. Future studies should also evaluate educational programs in terms of content, timing, teaching methods, and teacher qualifications.

Previous research results and our study results draw attention to the intense need for education that will provide physiotherapy students with awareness and field readiness, especially regarding “basic terminology about these concepts” and “the relationship between these concepts, health, and professional roles.” In addition, awareness should be raised about eco-friendly materials and creating a green clinic environment. The development of literacy skills regarding how to access basic informational resources should also be addressed. These topics should be incorporated into the entire teaching plan as part of the clinical decision-making process. This will ensure that students actively participate and apply their knowledge during practical classes and clinical internships. Furthermore, students’ needs regarding the subject should be regularly assessed, and the curriculum should be monitored and updated accordingly^54,57^. It also recommends monitoring developments in this area and sharing good examples^54^.

The most significant barriers during the process of incorporating these concepts into the curriculum included the intensity of the curriculum^58–60^ and the low awareness/competence of educators^58,60^ Time and resource constraints were also cited as other reasons^60^. Additionally, the urgent need for curriculum-related regulations^55^. The importance of multidisciplinary collaboration^58,59^ and professional accreditation studies, professional organisations, educators, and students working together in curriculum development was emphasized^55,60^. In addition, both in our study and in previous studies, the rate of students remembering what they heard about the subject in the lessons was low, which suggests that not only the awareness and needs of the students but also the educators should be evaluated. Previous studies emphasize the importance of training educators working in clinical and school settings on this topic^58,59^. It is also stated that platforms should be created to ensure and sustain development among educators^54^. It should not be forgotten that this group plays an important role model function. The responsibilities of physiotherapists should be included in planning not only for students but also for the individuals they serve (both patients and healthy individuals). The issue of what qualifications educators should have should be addressed with the participation of accreditation associations. Peer education processes, which have been shown to be effective as a method of supporting learning in physiotherapy education^61^ also be taken into consideration. Attention should also be paid to the training of student leaders during the education process^58^.

Stone et al. point out that the learning environment is an important factor in the educational process. They recommend incorporating sustainable practices into simulated and real clinical environments^57^. Irlam et al. report that faculties should determine their ecological footprints, develop future plans, and publish them to achieve a zero carbon footprint by 2050. Faculties and educators should also take the lead in developing policies on this subject^54^. In this process, educational institutions, students, and educators should develop environments (e.g., application rooms, green clinics, and eco-friendly campuses) and policies designed to support environmental sustainability.

It is recommended to develop different teaching methods and materials for EP and integrate these topics into clinical education, student practices, and theoretical courses within the curriculum^38^. Studies should be made about the importance of getting student opinions on EP and involving them in the process^4^. The following research results, which examine the question of whether health students need education on the subject and what kind of learning plans are in place to meet this need, show that students basically want courses to be added to the curriculum and that over the years, they have begun to express their needs regarding the subjects and methods that need to be taught. Simsek and Erkin reported that 89.4% of nursing students wanted to participate in SD-related training^12^. On the other hand, Shehu and Shehu stated that 71.93% of medical students believed that a course on SDGs should be included in university curricula^6^. Ladhar et al. found that 77% of pharmacy students believed a course on climate change and environmental sustainability should be added to the curriculum^16^. Chi et al. found that 60.8% of physiotherapists believed that topics such as climate change and its effects on health should be integrated into physiotherapy education^24^. Niemi et al. emphasized that 63% of medical and nursing students believed the SDGs and the 2030 Agenda should be a significant part of their education^11^. Irlam et al. found that 62.5% of medical and health and rehabilitation sciences students recalled only a few courses on environmental sustainability and that 51.5% of them stated the current curriculum did not adequately address the topic^20^. Kaşıkçı et al. emphasize the importance of incorporating sustainable practices into educational programs for future health professionals to promote sustainable practices in personal and professional settings^14^. Borlu et al. reported that 39.7% of medical students said they wanted to include a course on SD and the SDGs in the educational program^8^.

While following the results of previous research, another important question is which subjects and in what ways students want to learn these subjects. Borlu et al. showed that the two main topics that medical students requested to be added to their courses were “the role of the doctor in this subject” (35.7%) and “the relationship of the subject with health” (34%)^8^. Gupta et al. reported the following preferences for sustainable healthcare education among medical students: 60% preferred it to be delivered during the preclinical and clinical years; 31% preferred online education; 26% preferred lectures; and 24% preferred small group work. The students indicated that they most frequently wanted to learn about these topics from healthcare professionals and about the impact of national health policies on climate change^7^. Ladhar et al. reported that 70% of pharmacy students preferred integration activities, such as small group activities and case studies^16^. Swärdh et al. emphasized that physiotherapy education programs should be organized in an action-oriented manner^26^.Wieërs et al. found that 64% of health students in the faculty of medicine would like to see SDGs integrated into existing modules, 23% would like to see it as an optional module, and 13% would like to see it as a mandatory module^10^. Irlam et al. determined that the learning methods preferred by students were, in order, facilitated group discussions (36%), peer discussions (32.2%), online interactive resources (31.1%), case studies (32.1%), and elective courses (29.5%). The topics they wanted to be added to the curriculum were, in order, the effects of climate change on health (including health promotion) (22.3%), sustainable lifestyles (15.2%), reducing the effects of climate change (12.5%), and reducing carbon footprints (11%)^20^. In our study, despite students feeling that their knowledge level was insufficient, the percentage of those who felt the need for training was approximately 50% (SD: 51.8% - EFP: 51.4%). That is, approximately 50% stated that they did not feel the need for education. According to the transtheoretical model, this indicates that they are in the early stages of this model and require intensive training to enhance their awareness. This is because, according to this model, it is only possible to progress between stages once a sufficient level of awareness has been achieved^62^. Furthermore, this situation underscores the importance of conducting qualitative research on this topic in addition to surveys. Such research allows us to understand the reasons behind people’s behavior. Encouraging individuals to explain their answers can reveal new findings that were not initially considered but are equally important.

The importance of integrating multidisciplinary and collaborative learning activities, as well as student-centered, community-based assessment methods, is emphasized^58,59^. According to Irlam et al., training should incorporate critical thinking, problem-solving, and systematic thinking activities and assessment methods^59^. Additionally, the importance of including role-playing activities, real patient stories, and interprofessional^53,59,60^ and multi-professional activities^59^ is highlighted. Cross-disciplinary webinars, campus workshops, and interprofessional problem-solving activities are also recommended. Methods that prepare students for real patient experiences in a clinical setting at various stages, from taking a patient’s history to maintaining and treating their health, are explained. The concept of eco-ethical leadership is also emphasized. This process will be sustained in conjunction with national and international accreditation efforts^59^. In terms of the use of different methods, such as online/face-to-face, preclinical/clinical periods, compulsory/elective courses, regulations should be made in collaboration with professional accreditation associations/societies, professional organisations, educational institutions, educators, students, and other related disciplines. These regulations should be spread across the entire curriculum or added as a course. Active student participation should be a fundamental principle throughout the process.

Identifying factors that may influence students’ awareness is important for educational planning. In this regard, research has often focused on gender and class factors. Gupta et al. stated that the gender effect should be examined when planning sustainable healthcare education and that this factor should be taken into consideration during the creation of educational programs^7^. On the other hand, Yavaş et al. have stated that gender does not affect the carbon footprint of medical students^9^. Borlu et al. reported that medical students’ scores on the subscales of knowledge, attitude, and behavior related to sustainability were higher among female students^8^. Şimşek and Erkin found that female nursing students had higher scores on the total and subscales. However, no difference in scores was observed between class levels^12^. , Dönmez and Yardımcı. reported that nursing students’ scores were affected by “gender and cohabitants” factors (female > male; living with family > living with friends or living alone) but not by “age and class” factors^13^.

Dönmez and Yardımcı also reported that ‘place of residence for the longest period of time and perceived income level’ influenced the results of the sustainable consumption behaviour scale (those who lived with family, lived in metropolitan cities and reported low income level had higher scale results)^13^. Irlam et al. stated that there was no difference between classes and departments in terms of students’ understanding of climate change and environmental sustainability concepts^20^. Similarly, Niemi et al. showed that there was no difference in the general level of knowledge about SDGs and Agenda 2030 concepts and in the impact of SDGs on their future careers, as assessed by the medical and nursing students themselves, in their study^11^.

In our study, females tended to have higher scores for both concepts compared to males, while scores were similar across grades. In addition, females found using a greater number of sources of information for EFP compared to males. All these results and our research results were in line with the results of the research that women have a higher sensitivity to sustainability^43,63^. However, although the class effect has not been demonstrated in literature on awareness, our study also revealed that the number of resources used is affected by gender and class level. Similarly, Gunduz Ilgın et al. stated that gender and class level factors affect the resource choices of physiotherapy students, while only gender factors affect their subject preferences^64^. Therefore, when planning educational programs and making materials available to students, it is recommended that gender and class level factors be taken into account. Additionally, the effects of factors such as economic level, cohabitation status, and place of residence should be examined among physiotherapy students.

The EP Agenda plans to integrate EP into physiotherapy education by 2023, emphasizing its importance for future environmental initiatives in the profession. It is stated that understanding students’ backgrounds in secondary education can influence processes in tertiary education^38^. Previous studies evaluating students studying in the field of health showed that 37% and 98.8% of pharmacy students received education about the concepts of environmental sustainability^15,16^, planetary health^15^ and/or climate change^15^ in the period before their pharmacy education, respectively. The rates of encountering this information during pharmacy/dentistry education were 21%^16^ and 5%^17^, respectively. On the other hand, Borlu et al. emphasised that the SD knowledge level sub-score of medical school students was affected by the type of school where high school education was received and therefore, the education to be given about SD should be planned to cover all stages of education^8^. Similar to our study, it was observed that first-year students who had prior “environmental and health education” in secondary school had higher knowledge levels and used more information sources. These findings suggest that environmental education at each educational level may be related to each other, and emphasise the importance of taking into account the training received at previous educational levels when planning training for the target occupational group. In addition, in the study by Chi et al. 77.2% of respondents indicated that continuing education in the field of climate change and health was the most important solution^24^. Therefore, considering that each stage of education can influence other stages, this topic should be included in undergraduate, graduate, and in-service training curricula in the field of physiotherapy and rehabilitation.

Despite research indicating a high need for education, there are only a limited number of studies on the sources students use to access information. In a study by Irlam et al., where 264 health-related students (including 14 physiotherapy students) were analyzed, students frequently used the internet (84.1%) and social media (77.3%) as information sources. Other sources included lectures (31.4%) and scientific journals (31.4%)^20^. Borlu et al. reported that the most important sources of information about SD for medical students were social media, mass media, pre-faculty education and medical school education (42.3%, 24.5%, 14.3%, and 13%), respectively^8^. Shehu and Shehu stated that they frequently used the internet (41.35%) and television (16.75%) about SD goals, and the least frequent was the conference with a rate of 1.48%^6^. Previous research results once again highlight social media^64–68^ as the most important source of information preferred by health students during the COVID-19 period. Similarly, in our study, the internet and social media were the most preferred sources for both concepts. In addition to the disadvantages of social media use such as social media addiction, misinformation transfer and membership fees, it can be counted among the advantages that it provides students with a judgement free learning environment^69^, facilitates collaboration and supports visual learning^70^. Paying attention to these features in education and educational material planning should be considered as a factor that facilitates access to a wide range of students. It is essential for students to actively participate in this process. In addition to encouraging participation through student leaders and awards^54^, it will also be possible to engage a wider audience through online platforms.

On the other hand, students’ need for reliable and easily accessible sources of information is also intense. Also, it is recommended that special groups be formed for resource development^54^. However, there are studies showing that the use of scientific resources is not as common as the use of other resources.Gunduz Ilgın et al. showed that physiotherapy students only used health-related search engines for information about COVID-19 at a rate of 13.3% in their fourth year^64^. Irlam et al. showed that only 31.4% reported using scientific journals as a source^20^. Similarly, Shehu and Shehu stated that they used conferences as an information source at a rate of 1.48%^6^. In our study, we also found that students preferred to obtain less information about the subject from professional organizations, scientific publications, and scientific meetings. These results once again highlight the importance of creating scientific, reliable, up-to-date, economical, and environmentally friendly information sources/educational materials in collaboration with educational institutions, accreditation associations, professional organizations, and student participation, and making them available through open access to prevent information pollution. It highlights the need for national or international online platforms such as EPA worldwide from a professional perspective. In addition, both the intensive use of social media/internet resources and the needs that arose during the pandemic, as reported by Stone et al., bring to mind the need to be active in terms of resource development in areas such as tele-health and to be prepared to develop different types of resources that can be used with teleconferencing, video communication, and mobile phones^57^. In teaching plans, students’ readiness for these methods and their social media literacy competencies should be developed.

Professional organizations play an important role in raising awareness about these concepts and putting them into practice. In this regard, the EP Association holds quarterly meetings and shares open-access materials to meet the needs of students and physiotherapists for reliable and valid information on EP^71^. World Physiotherapy is a nonprofit organization registered as a charity (World Confederation for Physical Therapy) in the UK. World Physiotherapy states that physiotherapists should promote sustainability and health in their professional practice. According to reports, raising awareness of climate change and the factors influencing it will lead to positive changes^72^. The European Region of World Physiotherapy is a nonprofit, nongovernmental organization representing the physiotherapy profession at the European level. In May 2024, the European Region of World Physiotherapy published the working group’s basic recommendations for terminology and actions that physiotherapists should take in the field of sustainability. The 2030 SDGs highlight the importance of physiotherapists in achieving these goals^73^. However, in the research conducted and in our study, it is seen that students often prefer social media sources. Chi et al. found that Australian physiotherapists frequently cited the following organizations as reliable sources of information about climate change: the Commonwealth Scientific and Industrial Research Organization Climate Science Centre (84.6%), the World Health Organization (75.7%), the United Nations Framework Convention on Climate Change and the United Nations Environment Programme (75.6%), and the Bureau of Meteorology, Australia (75.6%). While 39.8% of physiotherapists thought the EPA was a reliable source, 30.8% stated that they were not familiar with the EPA. The Australian Physiotherapy Association was accepted as a reliable source by 56.4% of physiotherapists. In addition, 77.2% of physiotherapists indicated that statements published by professional associations are necessary, 77.2% indicated that educational materials for patients should be prepared, and 70.9% indicated that they believe the Australian Physiotherapy Association plays a very important advocacy role in this regard^24^. Sanchez-Ibáñez et al. indicate that 11.5% of people have heard of EPA^52^. Tonak and Kitis found that DPR students rarely used the World Confederation for Physical Therapy website (64.6%) and the internet search engines PubMed and PEDro (12.3%)^68^. This research shows that students need the support of educational institutions and professional organizations in their search for knowledge. It is crucial that national and international professional organizations contribute to information sharing by creating educational materials and providing access to reliable resources before and after graduation. Additionally, professional organizations play a crucial role in creating and disseminating statements on the subject^54^. Furthermore, the importance of national and international professional associations drawing students’ attention to the reliable resources available to them has once again been emphasized.

### Limitations

Our study has several limitations. Firstly, it is difficult to generalise because the data in the study are from DPR students at a single university. Secondly, when interpreting the results of this study, it is important to consider the potential limitations of self-report questionnaires, the results of which may be affected by reporting bias. Furthermore, despite the high awareness scores in our study and previous research, the high level of inadequacy felt by students suggests that the questionnaires used were not designed for healthcare professionals. This is because there is currently no specific questionnaire for the physiotherapy and rehabilitation field to assess awareness of professional practices and the connection between SD and EFP. On the other hand, previous studies have also emphasized the need for qualitative research in this field^57,74^. Thus, the factor that more in-depth data can be obtained not only through questionnaire surveys but also through qualitative research should also be taken into consideration in analysing the awareness about the subject and the needs in the field of education. Thirdly, the fact that the first and second part of data collection form used in this study was not evaluated in terms of face (Cohen’s Kappa Index and Fleiss’ kappa value), content, criterion-related and construct validity can be considered as another limitation of the study. However, it should be kept in mind that the percentage of agreement, which is an important indicator in terms of face validity, was aimed to be 100% between the researchers (Two researchers were working as academics; they had 25 and 8 years of experience, respectively, and one researcher was teaching an environmental and health course.) for each question and the final form was used after being reshaped according to the form editing suggestions. Also, during the interpretation of the data, the fact that it is the first study to provide data on the subject should be taken into consideration. Lastly, it is stated that physiotherapists are an important professional group in terms of sustainable health service delivery and in solving public health problems in the fields of reducing ecological footprint and combating climate change^75^. In this transformation process in terms of sustainable health care, it is recommended that education, research and practice areas should be taken into consideration on behalf of physiotherapy^76^. In this regard, we only evaluated the students who will be the professionals of the future. It will be important to reveal and compare the attitudes of physiotherapists working in clinical, research and educational fields in future studies.

## Conclusions

As a result, students in the physiotherapy and rehabilitation department have low awareness of the concepts of SD and EFP in terms of their professional roles. Previous studies conducted in other professional groups, as well as the results of this study, highlight the importance of translating the sense of inadequacy identified in this study from a theoretical concept in the field of physiotherapy and rehabilitation into clinical skills and attitudes. Additionally, our findings suggest that the following issues should be addressed during curriculum updates: These include the relationship and continuity between educational levels and models; educational content; assessment and teaching techniques; the teaching environment; educator competence; the process of preparing and sharing educational materials and information sources; active student participation; participation of professional organizations in the process; alignment with accreditation efforts; national and international collaboration; and examination of factors such as gender and class level that may influence these aspects.

## Electronic supplementary material

Below is the link to the electronic supplementary material.

Supplementary Material 1

Supplementary Material 2

## Data Availability

The data used during the current study are available from the corresponding author on reasonable request.

## Abbreviations

DPR: Department of physiotherapy and rehabilitation
EFAQ: Ecological footprint awareness questionnaire
EFP: Ecological footprint
EP: Environmental physiotherapy
HSF: Health sciences faculty
MCBU: Manisa Celal Bayar University
SD: Sustainable development
SDAQ: Sustainable development awareness questionnaire
SDGs: Sustainable development goals
