# Supplementary material for: Investigating awareness of the sustainable development and ecological footprint among physiotherapy and rehabilitation students
Source: Sci Rep. 2025 Jul 9;15:24740. doi: 10.1038/s41598-025-10399-y (PMC12241617; doi:10.1038/s41598-025-10399-y)
Supplement: Supplementary file 1 — Supplementary Material 1 [file 41598_2025_10399_MOESM1_ESM.docx]

| **Question Agreement Metrics** | | | | | | | | | | | | | | | | | | | | | | | | |
| --- | --- | --- | --- | --- | --- | --- | --- | --- | --- | --- | --- | --- | --- | --- | --- | --- | --- | --- | --- | --- | --- | --- | --- | --- |
| **Question Subject** | **Sociodemographic Characteristics?** | | | | | | | | **Views on sustainable development and ecological footprints** | | | | | | | | | | | | | | | |
| **Question Number** | | | | | | | | | | | | | | | | | | | | | | | | |
| **Questions** | **1** | **2** | **3** | **4** | **5** | **6** | **7** | **8** | **1** | **2** | **3** | **4** | **5** | **6** | **7** | **8** | **9** | | **10** | **11** | **12** | **13** | **14** | **15** |
| **Researcher Number (1/2)*** | | | | | | | | | | | | | | | | | | | | | | | | |
| **+= Yes; -= No** | | | | | | | | | | | | | | | | | | | | | | | | |
| Appropriateness of grammar | +/+ | +/+ | +/+ | +/+ | +/+ | +/+ | +/+ | +/+ | +/+ | +/+ | +/+ | +/+ | +/+ | +/+ | +/+ | +/+ | +/+ | | +/+ | +/+ | +/+ | +/+ | +/+ | +/+ |
| The clarity and unambiguity of items | +/+ | +/+ | +/+ | +/+ | +/+ | +/+ | +/+ | +/+ | +/+ | +/+ | +/+ | +/+ | +/+ | +/+ | +/+ | +/+ | +/+ | | +/+ | +/+ | +/+ | +/+ | +/+ | +/+ |
| The correct spelling of words | +/+ | +/+ | +/+ | +/+ | +/+ | +/+ | +/+ | +/+ | +/+ | +/+ | +/+ | +/+ | +/+ | +/+ | +/+ | +/+ | +/+ | | +/+ | +/+ | +/+ | +/+ | +/+ | +/+ |
| The correct structuring of the sentences | +/+ | +/+ | +/+ | +/+ | +/+ | +/+ | +/+ | +/+ | +/+ | +/+ | +/+ | +/+ | +/+ | +/+ | +/+ | +/+ | +/+ | | +/+ | +/+ | +/+ | +/+ | +/+ | +/+ |
| Appropriateness of font size and space | +/+ | +/+ | +/+ | +/+ | +/+ | +/+ | +/+ | +/-: For the internship status: The words “did” and “did not” should be capitalized, while the words “distance learning” and “face-to-face” should be written in lowercase. | +/ -: The phrases “sustainable development” and “ecological footprint” should be highlighted in capital letters and bold type. | | | | | | | | | | | | | | | |
| Legible printout | +/+ | +/+ | +/+ | +/+ | +/+ | +/+ | +/+ | +/+ | +/ -All questions should be placed in a table with a similar format, and section headings should be added. Table borders that are not bold should be used for printing purposes. | | | | | | | | | | | | | | | |
| Adequacy of instruction on the instrument | +/+ | +/+ | +/+ | +/+ | +/+ | +/+ | +/+ | +/+ | +/+ | +/+ | +/+ | +/+ | +/+ | +/+ | +/+ | +/+ | +/+ | | +/+ | +/+ | +/+ | +/+ | +/+ | +/+ |
| The structure of the instrument in terms of construction and well- thought out format | +/+ | +/+ | +/+ | +/+ | +/+ | +/+ | +/+ | +/+ | -: Questions 1-15= First, questions should be asked about the concept of sustainable development. Questions about the concept of ecological footprint should be asked second. / + | | | | | | | | | | | | | | | |
|  |  |  |  |  |  |  |  |  | +/+ | +/+ | +/+ | +/+ | +/+ | +/+ | +/ -:Information sources should be ranked according to their specific characteristics. | +/+ | +/+ | +/+ | | +/+ | +/+ | +/+ | +/ -: Information sources should be ranked according to their specific characteristics. | +/+ |
| Appropriateness of difficulty level of the instrument for the participants | +/+ | +/+ | +/+ | +/+ | +/+ | +/+ | +/+ | +/+ | +/+ | +/+ | +/+ | +/+ | +/+ | +/+ | +/+ | +/+ | +/+ | | +/+ | +/+ | +/+ | +/+ | +/+ | +/+ |
| Reasonableness of items in relation to the supposed purpose of the instrument | +/+ | +/+ | +/+ | +/+ | +/+ | +/+ | +/+ | +/+ | +/+ | +/+ | +/+ | +/+ | +/+ | +/+ | +/+ | +/+ | +/+ | | +/+ | +/+ | +/+ | +/+ | +/+ | +/+ |
| Corrections per question | Question 8 was reworded in line with the suggestion.  The words “did” and “did not” were capitalized.  The words “distance learning and face-to-face” were written in lowercase. | | | | | | | | Questions 1-15 and 7-14 were reorganized.  For the questions 1-15:   - The words “sustainable development” and “ecological footprint” were written in capital letters and bold type. - All questions were placed in a table. Section headings were added, and dark colors were not used for the table borders. - Throughout the form, questions about sustainable development were asked first, followed by questions about the ecological footprint.   For the qestions 7 and 14:   - Information sources with similar characteristics were listed consecutively. | | | | | | | | | | | | | | | |
| Post editing | | | | | | | | | | | | | | | | | | | | | | | | |
| % of per Question Agreement and  % of Overall Agreement | **100%** | | | | | | | | **100%** | | | | | | | | | | | | | | | |

*: Graduated from Physiotherapy and Rehabilitation Department (4 year), 25 and 8 years experienced. as physiotherapist/academician/researcher on physiotherapy and rehabilitation field and one researcher was teaching an environmental and health course.
