# Supplementary material for: Investigating awareness of the sustainable development and ecological footprint among physiotherapy and rehabilitation students
Source: Sci Rep. 2025 Jul 9;15:24740. doi: 10.1038/s41598-025-10399-y (PMC12241617; doi:10.1038/s41598-025-10399-y)
Supplement: Supplementary file 2 — Supplementary Material 2 [file 41598_2025_10399_MOESM2_ESM.docx]

**Supplementary Table 1: Sustainable development and ecological footprint awareness level according to grade.**

|  | **Grade** | **Mean** | **Standard Deviation** | **Median** | **Minimum** | **Maximum** | **p*** |
| --- | --- | --- | --- | --- | --- | --- | --- |
| **EFP-Food** | **1** | 3.0735 | 0.55996 | 3 | 2.13 | 5 | 0.065 |
|  | **2** | 3.0857 | 0.52936 | 3.125 | 1.50 | 4.38 |  |
|  | **3** | 2.9329 | 0.58963 | 3 | 1.13 | 4.5 |  |
|  | **4** | 3.1303 | 0.51795 | 3 | 1.63 | 4.75 |  |
| **EFP-Transportation/Housing** | **1** | 3.2268 | 0.71714 | 3.2143 | 1.57 | 5 | 0.469 |
|  | **2** | 3.2503 | 0.68568 | 3.2857 | 1.57 | 4.86 |  |
|  | **3** | 3.2153 | 0.70437 | 3.2143 | 1.29 | 5 |  |
|  | **4** | 3.3050 | 0.61498 | 3.2857 | 1.29 | 5 |  |
| **EFP-Energy** | **1** | 4.1060 | 0.66179 | 4.1667 | 1.33 | 5 | 0.192 |
|  | **2** | 4.1288 | 0.56427 | 4.1667 | 2 | 5 |  |
|  | **3** | 4.0245 | 0.66729 | 4 | 2 | 5 |  |
|  | **4** | 4.1974 | 0.61451 | 4.3333 | 2.25 | 5 |  |
| **EFP-Waste** | **1** | 3.9386 | 0.65793 | 3.9375 | 1.63 | 5 | 0.440^1^ |
|  | **2** | 3.8998 | 0.66254 | 3.8750 | 1.88 | 5 |  |
|  | **3** | 3.7996 | 0.79280 | 3.7500 | 1.5 | 5 |  |
|  | **4** | 3.8617 | 0.66927 | 3.8750 | 2.13 | 5 |  |
| **EFP-Water Consumption** | **1** | 3.9684 | 0.70777 | 4 | 1 | 5 | 0.140 |
|  | **2** | 3.9273 | 0.74565 | 4 | 1.8 | 5 |  |
|  | **3** | 3.8074 | 0.73893 | 3.8 | 1.2 | 5 |  |
|  | **4** | 3.9660 | 0.79497 | 4 | 1.8 | 5 |  |
| **EFP-Total** | **1** | 3.6627 | 0.53655 | 3.6396 | 1.75 | 5 | 0.144^1^ |
|  | **2** | 3.6584 | 0.47722 | 3.6405 | 2 | 4.59 |  |
|  | **3** | 3.5559 | 0.55721 | 3.6071 | 1.74 | 4.75 |  |
|  | **4** | 3.6921 | 0.48618 | 3.7021 | 2.14 | 4.59 |  |
| **SD-Economic Sustainability** | **1** | 55.6140 | 6.02724 | 57 | 24 | 64 | 0.069 |
|  | **2** | 55.0083 | 7.24856 | 56 | 17 | 64 |  |
|  | **3** | 53.7426 | 7.19156 | 54 | 23 | 65 |  |
|  | **4** | 55.3121 | 6.35962 | 56 | 31 | 65 |  |
| **SD-Social Sustainability** | **1** | 41.3246 | 4.69889 | 42 | 16 | 45 | 0.870 |
|  | **2** | 40.9008 | 5.97691 | 43 | 9 | 45 |  |
|  | **3** | 40.2279 | 6.14391 | 43 | 10 | 45 |  |
|  | **4** | 40.7730 | 5.26900 | 42 | 20 | 45 |  |
| **SD-Environmental Sustainability** | **1** | 61.0614 | 7.14240 | 63 | 27 | 70 | 0.169 |
|  | **2** | 59.9504 | 9.04328 | 62 | 17 | 70 |  |
|  | **3** | 59.1176 | 8.50887 | 61 | 25 | 70 |  |
|  | **4** | 60.9574 | 7.56861 | 62 | 36 | 70 |  |
| **SD-Total** | **1** | 158.0000 | 16.00553 | 162 | 68 | 178 | 0.195 |
|  | **2** | 155.8595 | 20.18882 | 161 | 48 | 178 |  |
|  | **3** | 153.0882 | 20.02608 | 157 | 64 | 178 |  |
|  | **4** | 157.0426 | 17.27627 | 161 | 87 | 180 |  |

SD: Sustainable Development; EFP: Ecological Footprint; *: p is for Kruskal-Wallis Analysis;  ^1^: p is for One Way ANOVA Analysis.
